# The Cajal body protein p80-coilin forms a complex with the adenovirus L4-22K protein and facilitates the nuclear export of adenovirus mRNA

**DOI:** 10.1128/mbio.01459-23

**Published:** 2023-10-05

**Authors:** Laura White, Bilgi Erbay, G. Eric Blair

**Affiliations:** 1 School of Molecular and Cellular Biology, Faculty of Biological Sciences, University of Leeds, Leeds, United Kingdom; Albert Einstein College of Medicine, Bronx, New York, USA; University of Pennsylvania Perelman School of Medicine, Philadelphia, Pennsylvania, USA

**Keywords:** adenovirus, early, intermediate and late viral transcripts, mRNA export, Cajal body, p80-coilin, RNA interference

## Abstract

Cajal bodies (CBs) are major sub-nuclear structures in most eucaryotic cells. In human adenovirus 5 (Ad5) infection, CBs are reorganized into microfoci in the late phase of infection. Here we show that many CB protein components (p80-coilin, SMN-1, and WRAP53) remained stable throughout most of the infectious cycle of Ad type 5 (Ad5) in human A549 epithelial cells, even when CBs were reorganized into microfoci. Reduction of p80-coilin expression by RNA interference resulted in significant reductions in the levels of early (E1A, E2A-DBP), intermediate (pIX and IVa2), and late (L1-IIIa, L2-penton base, L3-hexon, L4-100K, and L5-fiber) proteins in Ad5-infected A549 cells. Depletion of p80-coilin did not significantly alter the total cellular levels of the corresponding Ad cytoplasmic mRNAs (with the exception of E1A 12S and 13S and pIX mRNA) or the production of the Ad5 pre-mRNAs tested (E1A, E2A-DBP, IVa2, or late pre-mRNAs containing the tripartite leader). However, the ratio of viral cytoplasmic to nuclear-spliced Ad RNAs was reduced in p80-coilin-depleted, Ad5-infected cells compared to control-infected cells. Immunofluorescent staining of Ad5-infected cells revealed co-localization of p80-coilin with areas of immunoreactivity defined by a polyclonal antibody that recognized the L4-22K and L4-33K proteins in a fraction of microfoci. Immunoprecipitation analysis showed that only the L4-22K protein formed a stable complex with p80-coilin in Ad5-infected cells and in cells co-transfected with plasmids encoding p80-coilin and either the L4-22K or L4-33K protein. p80-coilin therefore plays an important role in Ad replication by complex formation with L4-22K and facilitating nuclear export of Ad mRNAs.

**IMPORTANCE:**

The architecture of sub-nuclear structures of eucaryotic cells is often changed during the infectious cycle of many animal and plant viruses. Cajal bodies (CBs) form a major sub-nuclear structure whose functions may include the regulation of cellular RNA metabolism. During the lifecycle of human adenovirus 5 (Ad5), CBs are reorganized from their spherical-like structure into smaller clusters termed microfoci. The mechanism of this reorganization and its significance for virus replication has yet to be established. Here we show that the major CB protein, p80-coilin, facilitates the nuclear export of Ad5 transcripts. Depletion of p80-coilin by RNA interference led to lowered levels of viral proteins and infectious virus. p80-coilin was found to form a complex with the viral L4-22K protein in Ad5-infected cells and in some reorganized microfoci. These findings assign a new role for p80-coilin as a potential regulator of infection by a human DNA virus.

## INTRODUCTION

Human adenoviruses (Ads) have attracted considerable interest as vaccine and gene therapy vectors, targeting diseases such as SARS-CoV2 coronavirus infection and human cancers as well as selective oncolytic agents (1, 2). Therefore, it is of great importance to understand the Ad lifecycle in human cells so that appropriate genetic modifications can be introduced to create viruses with therapeutic potential (2, 3). Ads are non-enveloped icosahedral viruses with a double-stranded DNA genome of approx. 36 kb encoding 30–40 proteins (4). Their lifecycle comprises three phases, namely, early, intermediate, and late, with the early and intermediate phases separated by the onset of viral DNA replication (1, 2). Early gene products promote cell cycle progression, genome replication, and the suppression of host cell anti-viral pathways (4). At the onset of DNA replication, intermediate transcription units are activated (5 – 7), and their gene products act to upregulate the expression of late genes that are transcribed from the major late promoter (MLP). Late proteins function mainly in the assembly and release of virus particles from the host cell.

Many plant and animal viruses target structures in the nucleus, presumably to facilitate the infection process (8, 9). During the late phase of Ad infection, there is a substantial reorganization of a sub-nuclear structure termed the Cajal body (CB) (10, 11). CBs are dynamic, non-membrane-bound structures involved in RNA metabolism (12 – 14). There are typically 1–6 CBs per nucleus, with their size and number being affected by the levels of splicing small nuclear ribonucleoproteins (snRNPs), cellular stress, and cell cycle stage (15 – 17). The major protein of CBs is p80-coilin, which appears to act as a scaffold to recruit other proteins to CBs, for example, survival of motor neuron protein 1 (SMN-1) and WD40-encoding RNA antisense to p53 (WRAP53) (18, 19). CBs have been detected in close proximity to sites of active transcription (20); however, as they lack native mRNA transcripts and contain inactive RNA polymerase II (21 – 23), they are believed to be involved in the assembly and regeneration of transcription and splicing complexes rather than being sites of active transcription or splicing (13). Interestingly, it has been proposed that CBs and their proteins may also play roles in DNA damage responses as well as snRNA processing and export (24 – 28).

During the late phase of Ad5 infection, CBs are disassembled into microfoci, which have also been termed “rosettes” (29). Here we show that the levels of the major CB proteins, p80-coilin and SMN-1, remained stable throughout most of the Ad5 infectious cycle in A549 epithelial cells, even at times when CBs were rearranged into microfoci. Depletion of p80-coilin in A549 cells by small interfering RNA (siRNA) treatment resulted in a significant decrease in virus yield and production of several early, intermediate, and late viral proteins compared to control siRNA-treated cells. Analysis of Ad5 mRNA levels by reverse transcription-quantitative PCR (RT-qPCR) revealed that, while the levels of most Ad5 mRNAs were unaffected by p80-coilin depletion, the ratio of spliced cytoplasmic to nuclear Ad mRNAs was decreased in p80-coilin-depleted cells. Immunofluorescent antibody staining and confocal microscopy analysis revealed that p80-coilin co-localized with areas of immunoreactivity defined by a polyclonal antibody raised against the viral splicing factor L4-33K in a fraction of microfoci in Ad5-infected cells. No co-localized immunoreactivity was observed with Aly, a component of the major mRNA export TREX complex. Further studies on the specificity of two independent polyclonal antibodies raised against purified L4-33K showed that these antibodies also recognized the related L4-22K protein, which shares an N-terminal domain with L4-33K but has a unique carboxy-terminal domain due to differential pre-mRNA splicing. Co-immunoprecipitation analysis revealed that p80-coilin formed a stable complex with L4-22K in Ad5-infected A549 cells; moreover, myc-tagged p80-coilin and FLAG-tagged L4-22K (but not FLAG-tagged L-33K) formed a stable complex in 293T cells co-transfected with expression plasmids encoding the viral proteins. Overall, these results suggest that the CB protein p80-coilin forms a stable complex with L4-22K and facilitates Ad mRNA transport throughout all phases of infection, pointing to a new role for the CB in Ad infection.

## RESULTS

### The major protein components of the CB remain stable throughout most of the Ad5 infectious cycle

**Fig 1:**
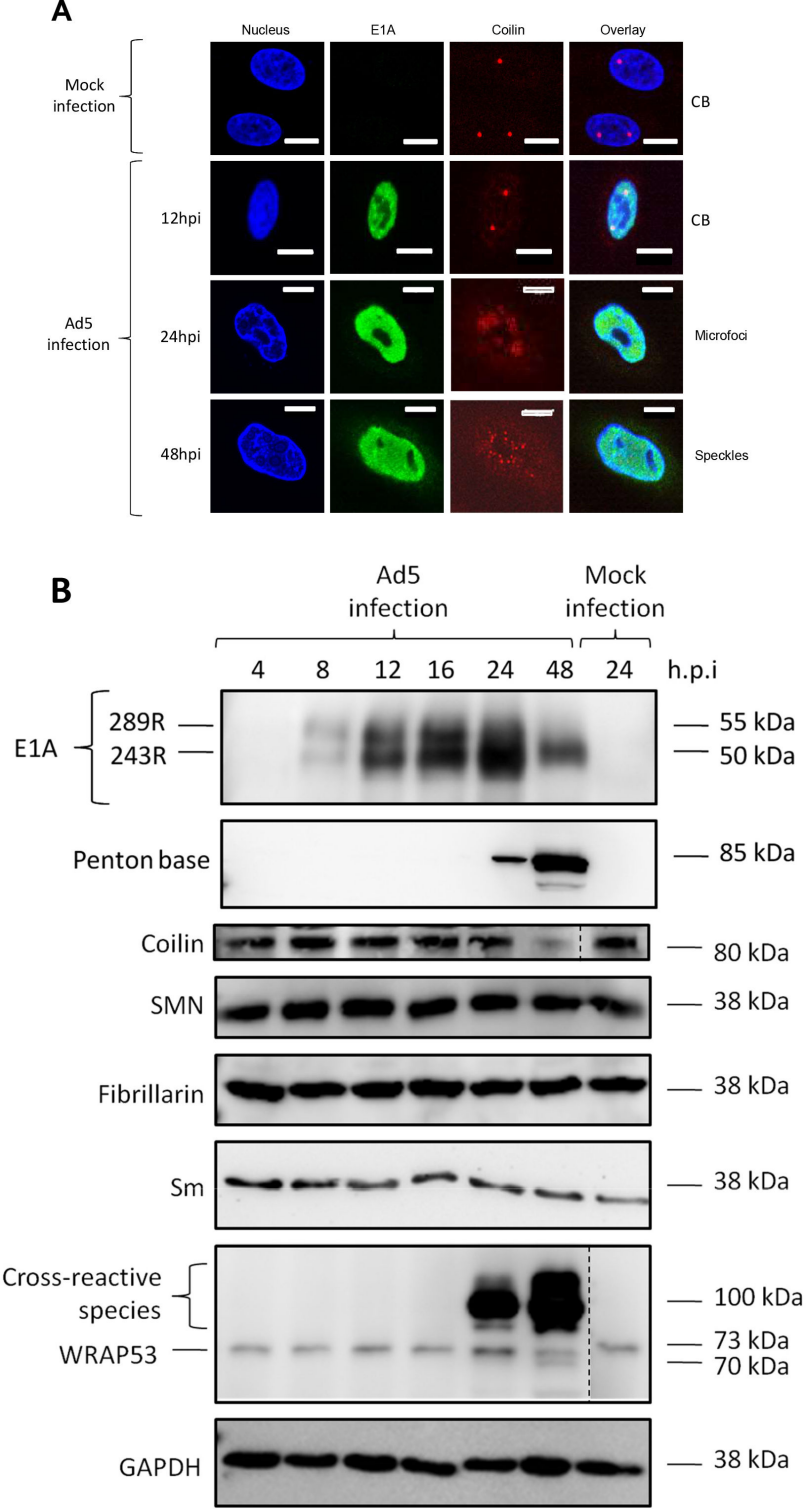
Cajal bodies (CBs) are reorganized during adenovirus 5 infection of A549 cells without major changes in the levels of CB-associated proteins. A549 cells were infected with Ad5 at an MOI of 5 FFU/cell and incubated for 0, 12, 18, 24, or 48 hours post-infection (hpi). (**A**) Indirect immunofluorescence and confocal microscopy were performed to locate p80-coilin (red) and Ad5 E1A proteins (green) in mock- and Ad5-infected cells. (**B**) Cell lysates were prepared, and equal masses of protein from each sample (20 µg) were analyzed by Western blotting using the indicated antibodies. Bound antibodies were detected by chemiluminescence. Blots were also quantitated by densitometric scanning to confirm the data shown (results not shown). The anti-E1A antibody detects the two major E1A proteins of 243 and 246 amino acid residues (R). The anti-WRAP53 antibody binds non-specifically to an approx. 100 kDa protein in Ad5-infected cells. Note also that in the anti-WRAP53 blot, the mock-infected control lane was derived from another position on the same blot, signified by the vertical broken line.

During the infectious cycle of Ad5, CBs are reorganized into microfoci (also termed “rosettes”) (10, 11, 29); however, it is not clear if the levels of CB-associated proteins are altered during this reorganization step. A549 cells were infected with Ad5, and immunofluorescent antibody staining and confocal microscopy were performed at 12, 24, and 48 hours post-infection (hpi). Cells were stained for p80-coilin, the viral immediate early E1A proteins (as a marker for Ad5-infected cells), and DAPI (to stain nuclei). This showed that CBs were reorganized into microfoci between 12 and 24 hpi, and by 48 hpi, CBs were further redistributed into nuclear speckles (Fig. 1A). In parallel, the levels of the CB proteins p80-coilin, SMN-1, WRAP53 as well as Sm (a component of spliceosomal snRNPs) and fibrillarin (a protein that shuttles between nucleoli and CBs) (30, 31) were analyzed by Western blotting (Fig. 1B). The levels of SMN-1, Sm, and fibrillarin proteins remained constant throughout infection. The cellular levels of p80-coilin and WRAP53 were stable up to 24 hpi, but were reduced by approx. 20 and 40%, respectively, by 48 hpi compared to mock-infected cells (Fig. 1B), at a time when microfoci were further reorganized into nuclear speckles (Fig. 1A). Markers for the early and late phases of Ad infection, the immediate early E1A proteins and the late penton base protein, were detected at 8 and 24 hpi, respectively.

### Depletion of p80-coilin from A549 cells did not affect cell viability but resulted in reduced production of Ad5 proteins and infectious virus

**Fig 2:**
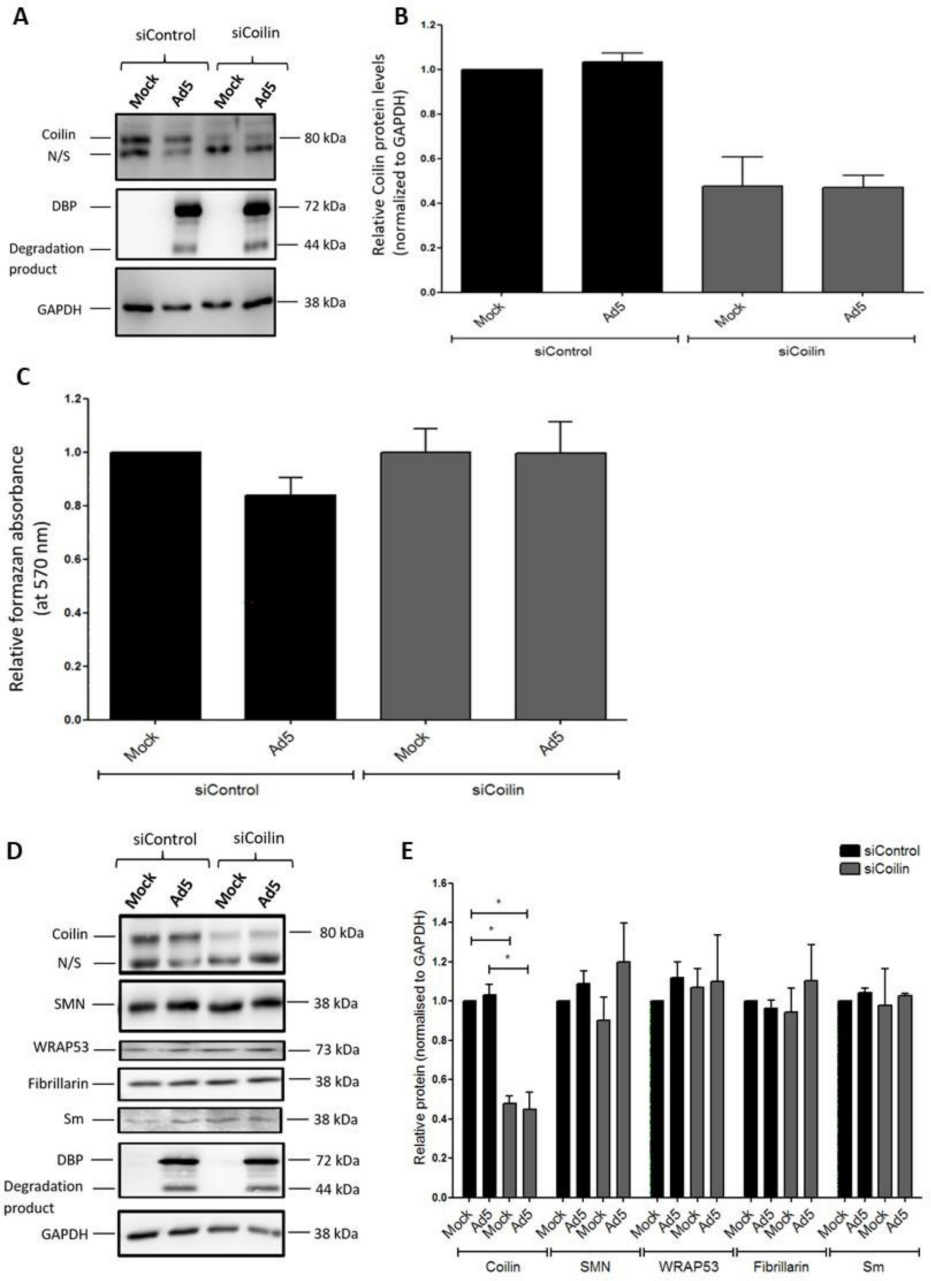
Reduction of p80-coilin by RNA interference in A549 cells does not alter cell viability or the steady-state level of other Cajal body-associated proteins. A549 cells were transfected with siRNAs specific for p80-coilin (siCoilin) or control All Stars siRNA (siControl) and incubated for 24 hours. Cells were either infected with Ad5 at an MOI of 5 FFU/cell or mock infected and incubated for a further 24 hours. (**A**) Whole-cell lysates were prepared, and equal masses of protein (20 µg) were analyzed by Western blotting using rabbit anti-p80-coilin, a mouse antibody against the viral E2A-72K DNA-binding protein (DBP) or a mouse anti-GAPDH antibody followed by incubation with the appropriate secondary HRP-conjugated antibody. Bound antibodies were detected by chemiluminescence. (**B**) p80-coilin protein levels were determined by densitometric analysis and normalized to the signal intensity of the loading control, GAPDH. Normalized p80-coilin levels are shown relative to the siControl-treated, mock-infected sample, which was set to a value of 1. Values are the mean (±SEM) from three independent experiments. (**C**) Cell viability assays were performed using an MTT assay following the treatment of A549 cells with p80-coilin or control siRNA followed by infection with Ad5, as described above. Values are the mean (±SEM) from three independent experiments. Data are displayed relative to the siControl-treated, mock-infected sample, which was set to a value of 1. No significant differences were found in cell viability between cells transfected with control or p80-coilin siRNA or mock- and Ad5-infected cells.(**D**) Western blot analysis of CB-associated protein levels in cells treated with control or p80-coilin siRNA followed by Ad5 infection. (**E**) Densitometric analysis of protein band intensities from Western blotting experiments shown in (**D**). Signal intensities were calculated by densitometric analysis using AIDA software and were normalized to the signal intensity of the internal loading control, GAPDH. Values are displayed relative to siControl-treated, mock-infected sample, which was set to a value of 1. Data are displayed as the mean (±SEM) from three independent experiments. * *P* ≤ 0.05. A non-specific (N/S) band was detected by the anti-coilin antibody.

In order to define a role for p80-coilin in Ad5 infection, depletion of p80-coilin in A549 cells was performed using p80-coilin siRNA (siCoilin), and cells were either mock- or Ad5-infected. The extent of p80-coilin depletion was determined in whole-cell lysates by Western blotting (Fig. 2A and B). Densitometric analysis revealed that p80-coilin levels were reduced in siCoilin-treated cells by 53% and 55% in mock- and Ad5-infected cells, respectively, compared to siControl-treated cells (Fig. 2B). There was no significant difference in the p80-coilin levels in siCoilin-treated mock-infected cells compared to siCoilin-treated Ad5-infected cells, indicating that Ad5 infection did not alter the efficacy of the siRNA treatment.

Depletion of p80-coilin has previously been suggested to result in a significant reduction in the proliferation of HeLa cells, although cells still appeared to be viable (32). To establish whether p80-coilin depletion altered the viability of A549 cells, MTT assays were performed (33). As shown in Fig. 2C, the viability of siCoilin-treated, mock-infected cells was not significantly altered compared to siControl-treated, mock-infected cells. The viability of siCoilin-treated, Ad5-infected cells was not significantly altered compared to siControl-treated, Ad5-infected cells, indicating that p80-coilin depletion did not alter the viability of Ad5-infected cells. Interestingly, p80-coilin depletion did not alter the steady-state levels of the other CB-associated proteins, SMN-1 and WRAP53, or of the nuclear proteins Sm and fibrillarin that shuttle between CBs and speckles or nucleoli, respectively (Fig. 2D and E).

**Fig 3:**
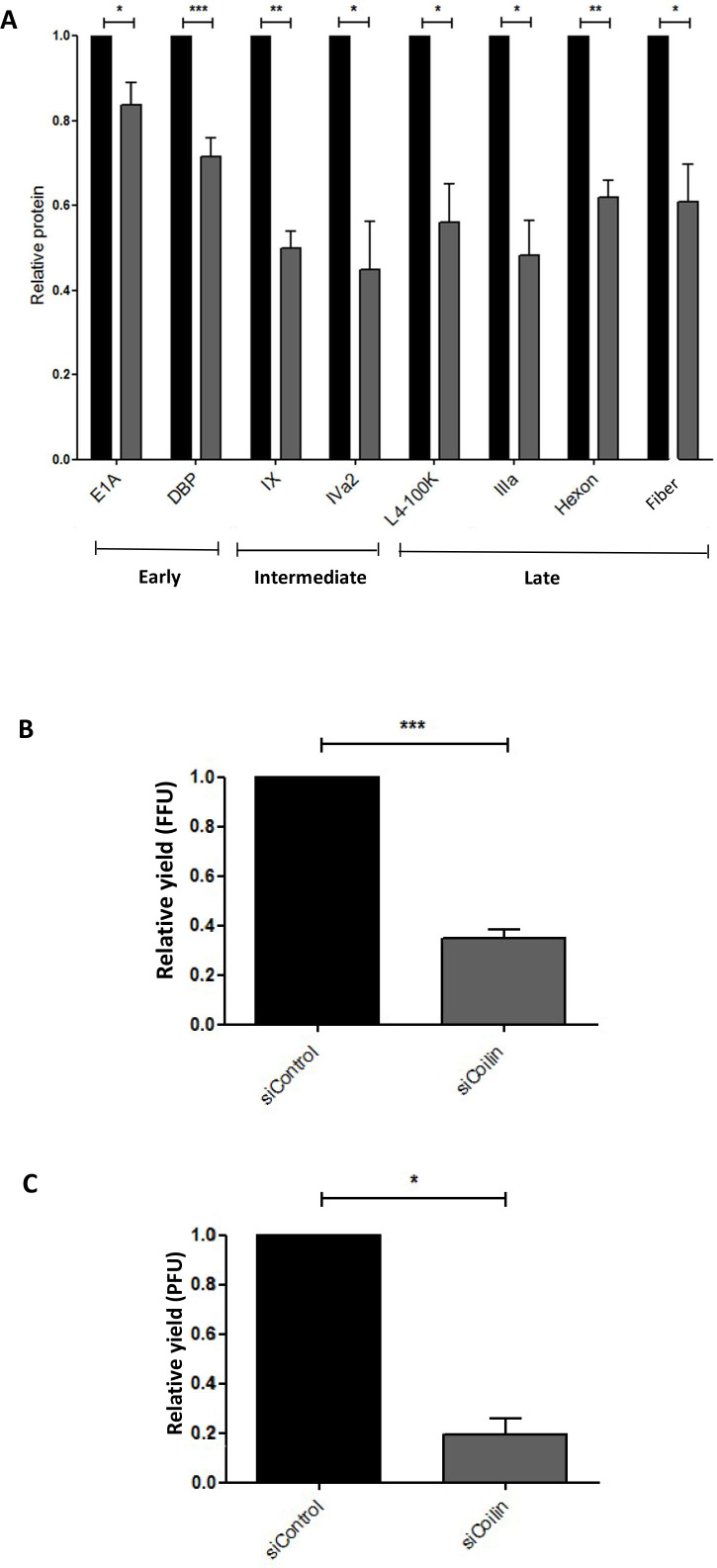
Depletion of p80-coilin leads to a reduction in the level of a range of viral proteins and infectious virus in adenovirus 5-infected A549 cells. (**A**) Flow cytometry analysis of viral proteins in control or p80-coilin siRNA-treated cells. A549 cells were transfected with siRNAs specific for p80-coilin (siCoilin) or control All Stars siRNA (siControl) and incubated for 24 hours. Cells were either infected with Ad5 at an MOI of 5 FFU/cell or mock infected, incubated for a further 24 hours, harvested, and analyzed for viral protein expression using specific antibodies for each viral protein by flow cytometry. Viral protein levels (as judged by geometric mean fluorescence units) were obtained from at least four independent experiments performed in duplicate and are shown relative to the control siRNA-treated, Ad5-infected sample, which was set to a value of 1, denoted by the black columns. The shaded columns denote viral protein level in p80-coilin siRNA-treated samples. Data show the mean ± SEM. Statistics were calculated using the paired one-sample *t*-test. *P < 0.05, **P < 0.01, ***P < 0.001. (**B**) Flow cytometry analysis of infectious virus produced in p80-coilin-depleted cells. Following infection of control or p80-coilin siRNA-treated A549 cells with Ad5, cell lysates were prepared and used to infect fresh A549 cells. At 48 hpi, cells were fixed and permeabilized, and the viral hexon protein (a marker for viral infection) was quantitated by flow cytometry, measuring geometric mean fluorescence units. Data were obtained from at least four independent experiments performed in duplicate and are shown relative to the control siRNA-treated, Ad5-infected sample, which was set to a value of 1, denoted by the black column. Virus production in p80-coilin siRNA-treated A549 cells is shown in the shaded column. ***P < 0.001 (**C**) Infectious Ad5 produced in A549 cells treated with either siControl or siCoilin siRNA determined by viral plaque assay (34). Cells were harvested, lysates were prepared, and 10-fold serial dilutions were used to infect fresh A549 cells that were incubated for six days before plaques were counted. The average virus titer following infection of A549 cells with cell lysate from siControl-treated, Ad5-infected cells was 1.5 × 10^8^ plaque-forming units (PFU)/mL. *P < 0.05.

The impact of p80-coilin depletion on Ad5 infection was investigated by analyzing the production of a range of Ad5 proteins, comprising the products of early, intermediate, and late genes. The levels of Ad5 early (E1A and E2A-DBP), intermediate (IVa2 and pIX), and late (L1-IIIa, L3-hexon, L4-100K, and L5-fiber) proteins were assayed by antibody staining of intracellular proteins and flow cytometry, following p80-coilin depletion and Ad5 infection of A549 cells. Levels of viral proteins were measured in geometric mean fluorescence units. As shown in Fig. 3A, flow cytometric analysis revealed a significant decrease in the levels of all Ad proteins tested following Ad5 infection of siCoilin-treated cells compared to Ad5 infection of siControl-treated cells. Interestingly, the levels of early proteins (E1A and E2A-DBP) were less extensively reduced compared to intermediate (pIX and IVa2) and late (L4-100K, L1-IIIa, L3-hexon and L5-fiber) proteins.

The effect of p80-coilin depletion by RNA interference on the production of infectious Ad5 was also investigated in mock- and Ad5-infected A549 cells. Following infection of siRNA-transfected cells, 10-fold serial dilutions of cell lysates were used to treat fresh A549 cells. After 24 hours incubation, cells were analyzed by intracellular antibody staining for the major capsid protein, L3-hexon, followed by flow cytometry. This showed that the production of Ad5 virus in siCoilin-treated Ad5-infected A549 cells was reduced by 80.4 ± 6.0% (*P* < 0.05) when compared to siControl-treated Ad5-infected cells, as measured by a fluorescent-forming focus assay (Fig. 3B). Similar results were obtained using a conventional virus plaque assay (Fig. 3C).

### Depletion of p80-coilin reduced Ad5 mRNA export from the nucleus to the cytoplasm

**Fig 4:**
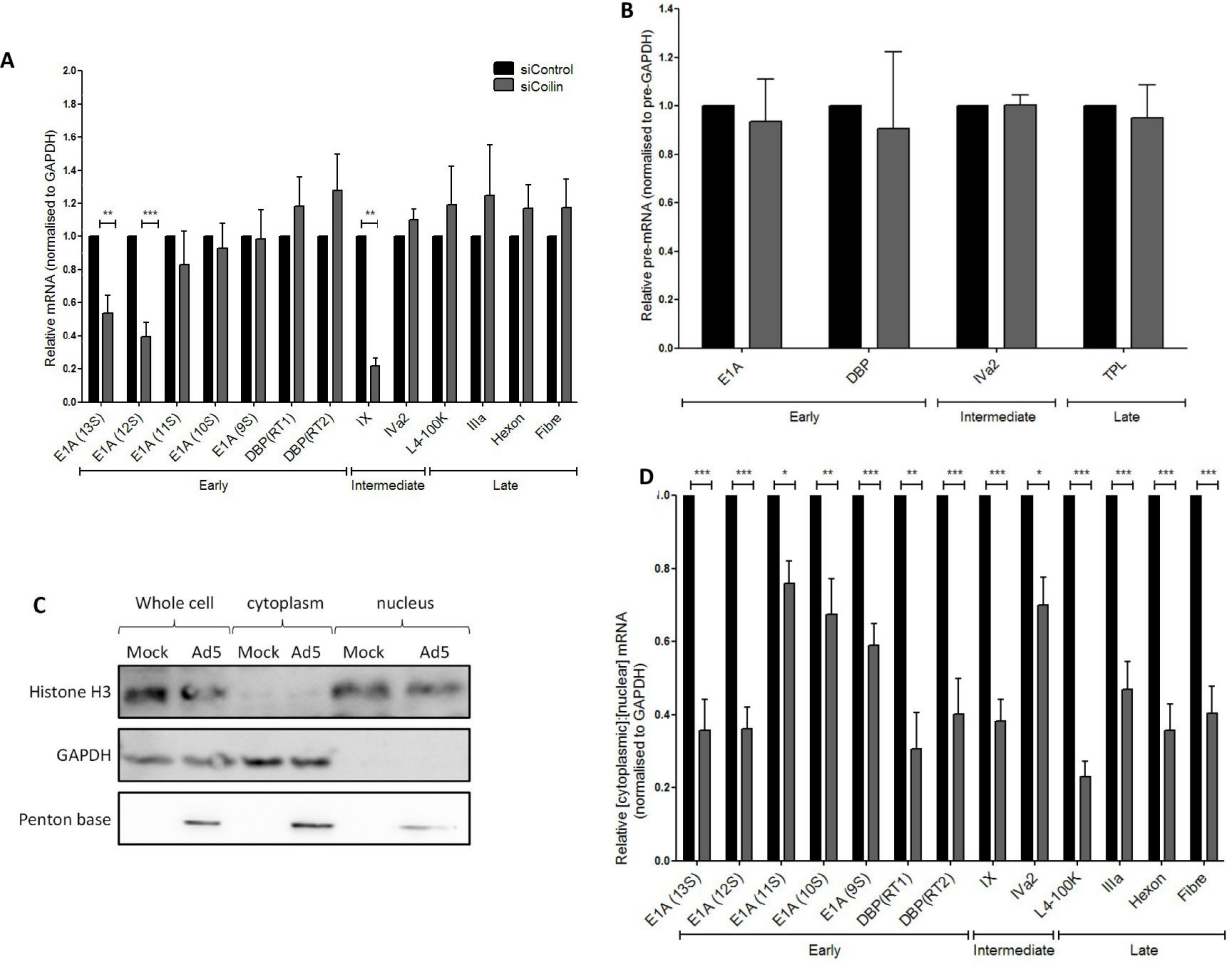
Nuclear export of viral mRNA in adenovirus 5-infected cells is reduced in p80-coilin-depleted A549 cells. Cells were harvested, total RNA was extracted by TRIzol-chloroform extraction, and levels of viral mRNAs were determined by quantitative PCR (qPCR) relative to the cellular GAPDH transcript. Data are displayed as relative to the siControl-treated, Ad5-infected sample, which was set to 1 (shown by the black columns). The relative levels of viral transcripts are denoted by the shaded columns. Results are shown as the mean (±SEM) from at least three independent experiments performed in duplicate. All statistics were calculated using the paired one-sample *t*-test. *P < 0.05, **P < 0.01, ***P < 0.001. (**A**) The levels of expression of Ad5 mRNAs present in total cellular RNA at 20 hpi following Ad5 infection of control or p80-coilin-treated A549 cells. (**B**) The expression levels of total cellular Ad5 pre-mRNAs following Ad5 infection of control or p80-coilin-depleted A549 cells. Quantitative RT-qPCR analysis was performed using exon-intron spanning primers specific for each pre-mRNA template. The results are displayed as the mean (±SEM) from at least three independent experiments performed in duplicate. The data are displayed as relative to the siControl-treated, Ad5-infected sample, which was set to a value of 1 (denoted by black columns). No statistically significant differences were found between levels of any viral pre-mRNA tested in control and p80-coilin-depleted A549 cells. (**C**) Western blot analysis of subcellular fractions to validate the purity of the nuclear and cytoplasmic fractions. A549 cells were treated with siControl or siCoilin and incubated for 24 hours. Cells were mock- or Ad5-infected at an MOI of 5 FFU/cell and incubated for 24 hours. Subcellular fractionation of mock- and Ad5-infected cells was performed, and whole-cell, nuclear, and cytoplasmic fractions were isolated. Equal masses of protein (20 µg) from each sample were separated by SDS-PAGE and analyzed by Western blotting. Histone H3 was used as a nuclear marker, GAPDH was used as a cytoplasmic marker, and Ad penton base was used as a marker of Ad5 infection. Whole-cell fractions contained both histone H3 and GAPDH. Nuclear fractions contained histone H3 but were negative for GAPDH. Cytoplasmic fractions contained GAPDH but were negative for histone H3. Penton base was detected in whole-cell, nuclear, and cytoplasmic fractions of Ad5-infected but not mock-infected cells. (**D**) The cytoplasmic:nuclear ratio of Ad5 mRNAs following Ad5-infection of p80-coilin-depleted A549 cells. Cells were separated into nuclear and cytoplasmic fractions. Total RNA was extracted from each fraction, quantitative RT-qPCR analysis of viral mRNAs was performed using exon-spanning primers specific for each mRNA target (except for pIX) and normalized to the level of the cellular GAPDH transcript. The cytoplasmic:nuclear ratio for each viral mRNA (normalized to GAPDH) was calculated. Results were obtained from at least three independent experiments with samples analyzed in duplicate and are shown as the mean (± SEM). Data are displayed relative to the siControl-treated Ad5-infected sample, which was set to a value of 1 (denoted by black columns).

To establish whether the observed decreases in Ad5 early, intermediate, and late proteins following p80-coilin depletion were due to decreased Ad5 mRNA levels, total cellular (nuclear plus cytoplasmic) RNA was isolated from siCoilin- or siControl-treated, Ad5-infected A549 cells, and reverse transcription-quantitative PCR (RT-qPCR) analysis was performed. The majority of Ad5 transcripts are generated by complex alternative RNA splicing. The primary E1A transcript is processed to yield five mRNAs which, in Ad5, have sedimentation coefficients of 13S, 12S, 11S, 10S, and 9S (33, 35). E2A-DBP mRNAs comprise two distinct species: the early expressed RT1, consisting of two short leader sequences and the main body of the mRNA, and the delayed early RT2, consisting of a single short leader sequence and the main body (36). Finally, transcription of late mRNAs such as L1-IIIa, L3-hexon, L4-100K, and L5-fiber is initiated from the major late promoter (MLP). The MLP drives the transcription of five families of late mRNA transcripts termed L1-5 that are differentially spliced and polyadenylated. Each mRNA is composed of an identical tripartite leader sequence of non-coding RNA at the 5′ end, generated by the removal of three introns and alternative splicing to the exons of the mRNA families (37 – 41). Exon-spanning primers were designed to amplify mature, spliced Ad5 mRNAs, namely, E1A isoforms 13S, 12S, 11S, 10S, and 9S, and E2A-DBP spliceoforms RT1 and RT2, IVa2, L1-IIIa, L3-hexon, L4-100K, and L5-fiber. The Ad pIX transcript does not undergo splicing (42); therefore, primers for this mRNA were designed within the single exon of pIX. The specificity of the designed primers and the size of the RT-PCR products derived from mock- and Ad5-infected A549 cells were established by gel electrophoresis (results not shown). No significant changes were detected by RT-qPCR in the levels of E1A 9S, E1A 10S, E1A 11S, E2A-DBP RT1, DBP RT2, IVa2, L1-IIIa, L3-hexon, L4-100K, and L5-fiber mRNA transcripts following Ad5 infection of siCoilin-treated A549 cells compared to Ad5 infection of siControl-treated cells (Fig. 4A). However, the levels of E1A 13S, E1A 12S, and pIX mRNA transcripts were significantly reduced following the Ad5 infection of siCoilin-treated cells compared to the Ad5 infection of siControl-treated cells (*P* < 0.05).

Ad5 pre-mRNAs were analyzed by RT-qPCR using intron-exon spanning primers. As shown in Fig. 4B, there were no significant alterations in the levels of E1A, E2A-DBP, IVa2, or tripartite leader (TPL)-containing pre-mRNAs following Ad5 infection of siCoilin-treated cells compared to Ad5 infection of siControl-treated cells. Overall, this indicated that p80-coilin depletion did not alter transcription from the E1A, E2A-DBP, IVa2, or major late transcriptional units.

Since there was no significant alteration in the level of most Ad5 mRNAs following p80-coilin depletion but there was a reduction in the levels of the corresponding Ad proteins (Fig. 3A), this suggested that p80-coilin may play a role at a post-transcriptional, post-RNA splicing level or at a translational or post-translational level. As p80-coilin is primarily a nuclear protein, its potential role in the process of viral mRNA export was investigated. Nuclear and cytoplasmic fractions were isolated from A549 cells following siRNA transfection and mock or Ad5 infection. The specificity of cell fractionation was established by Western blotting for protein markers of nuclei (histone H3) and cytoplasm (GAPDH) (Fig. 4C), confirming that the nuclear fraction had no detectable cytoplasmic contamination as judged by the lack of GAPDH signal in the Western blot. Conversely, the cytoplasmic fraction did not contain histone H3. As expected, the L2 penton base protein was present in nuclei, the site of virus assembly. RNA isolated from the cell fractions was analyzed by RT-qPCR and the ratio of cytoplasmic:nuclear mRNA for viral transcripts was calculated. An increase in this ratio would indicate an increase in the export of transcripts from the nucleus to the cytoplasm, whereas a decrease would be characteristic of reduced mRNA export. As shown in Fig. 4C, for all Ad transcripts studied, Ad5 infection of siCoilin-treated cells resulted in a significant decrease in the ratio of cytoplasmic: nuclear mRNA transcripts compared to infection of siControl-treated cells. This indicates that, following p80-coilin depletion, there is an accumulation of Ad5 mRNAs in the nucleus and reduced levels of Ad5 mRNAs in the cytoplasm, pointing to a role for p80-coilin in facilitating Ad5 mRNA export.

### Interaction of Ad5 proteins with CB microfoci and p80-coilin in infected cells

A major pathway of mRNA transport into the cytoplasm involves the TREX complex, which is recruited to nascent mRNA in a transcription- and splicing-dependent manner (43). Therefore, the potential association of p80-coilin with the TREX complex in Ad5-infected cells was assessed by immunofluorescent antibody staining and confocal microscopy using a rabbit anti-p80-coilin antibody and a mouse antibody raised against a component of the TREX complex, Aly (43). A goat anti-Ad capsid antibody was used to identify Ad5-infected cells.

**Fig 5 F5:**
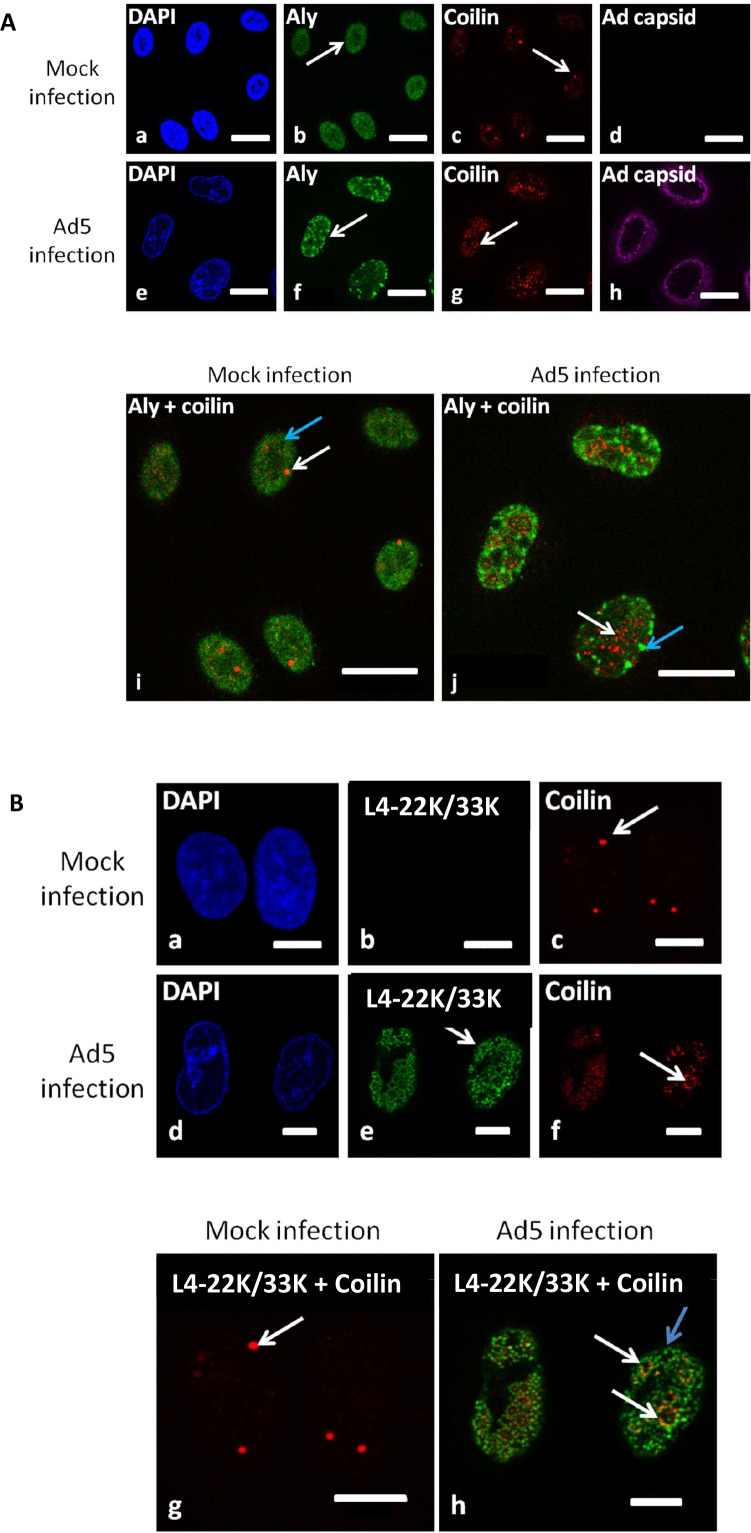
p80-coilin co-localizes with areas in microfoci that are stained with an antibody that recognizes L4-33K and L4-22K, but does not co-localize with the TREX mRNA export complex in Ad5-infected cells. A549 cells were mock-treated or infected with Ad5 at an MOI of 5 FFU/cell and incubated for 24 hours. (**A**) Cells were fixed, stained with a mouse anti-Aly antibody (specific for the TREX complex) and a rabbit anti-p80-coilin antibody followed by an Alexa Fluor-488-labeled goat anti-mouse immunoglobulin (shown in green) and an Alexa Fluor-594-labeled goat anti-rabbit immunoglobulin (shown in red). In addition, Ad5-infected cells were identified using a goat anti-Ad5 capsid antibody, followed by Alexa Fluor-647-labeled donkey anti-goat immunoglobulin (shown in purple). Nuclei were stained using DAPI. Images were obtained by confocal microscopy. In panels a to g, white arrows denote specifically labeled cells. in the overlaid images in panels i and j, white arrows denote p80-coilin and blue arrows indicate Aly. White bars are equivalent to 20 µm. (**B**) Cells were fixed, stained with a mouse anti-p80-coilin antibody and a rabbit antibody raised against L4-33K (that also recognizes L4-22K) followed by incubation with an Alexa Fluor-594-labeled goat anti-mouse immunoglobulin (shown in red) and an Alexa Fluor-488-labeled goat anti-rabbit immunoglobulin (shown in green), and examined by confocal microscopy. Nuclei were stained using DAPI. In panels a to f, white arrows show specifically labeled cells. In the overlaid images in panels g and h, white arrows show p80-coilin and the blue arrow shows anti-L4-22K/33K antibody labeling. Note areas of co-localized staining by the anti-L4-22K/33K antibody and p80-coilin antibodies as shown by the white arrows in panel h. White bars are equivalent to 10 µm.

As shown in Fig. 5, p80-coilin was located in punctate CBs in mock-infected cells (Fig. 5A,c and B,c [Fig F5] [Fig F5]). Aly was located in separate nucleoplasmic speckles, with exclusion from large rounded structures within the nucleus that are likely to be nucleoli (Fig. 5A,b). In Ad5-infected cells, p80-coilin was redistributed into microfoci, and Aly was reorganized into punctate nucleoplasmic structures (Fig. 5A,f). However, there did not appear to be any co-localization between p80-coilin and Aly (Fig. 5A,j). This indicates that p80-coilin does not associate with the TREX complex during Ad5 infection.

The possibility that an adenovirus protein might localize with CB microfoci was explored by using immunofluorescence to screen a panel of antibodies raised against a range of Ad5 proteins including the antibodies used to determine viral protein levels in Fig. 3A and a polyclonal rabbit antibody raised against L4-33K derived from Ad-infected cells. Only the L4-33K antibody recognized microfoci in Ad5-infected cells (44). This showed a punctate nucleoplasmic distribution (Fig. 5B, e) and co-localized with p80-coilin in a fraction of microfoci, shown by the yellow regions in the overlay (Fig. 5B,h).

**Fig 6:**
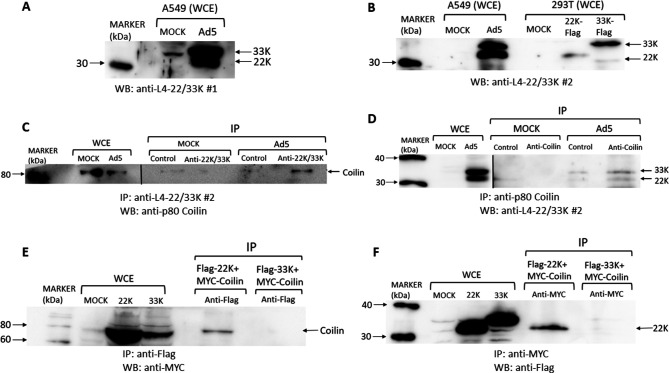
Molecular interactions between the adenovirus 5 L4-22K and 33K proteins and p80-coilin. (**A,B**) A549 cells were mock-treated or infected with Ad5 at a MOI of 5 FFU/cell and incubated for 24 hours. Whole-cell extracts (WCE) were prepared as described in Materials and Methods for Western blotting; 20 µg protein was analyzed by SDS-PAGE and Western blotting with the indicated antibodies. In (**B**), a further experiment was performed in which 293T cells were transfected with plasmids designed to express Flag-tagged L4-22K or 33K or were mock-transfected. WCE were analyzed by Western blotting using rabbit anti-L4-22K/33K serum. (**C,D**) A549 cells were mock-treated or infected with Ad5 at a MOI of 5 FFU/cell and incubated for 24 hours. WCE were prepared as described in Materials and Methods for immunoprecipitation (IP), using protease and phosphatase inhibitors in the lysis buffer. In (**C**), 100 µg protein was immunoprecipitated with rabbit anti-L4-22K/33K, as described in Materials and Methods. Samples of the WCE were also analyzed to confirm the expression of p80-coilin. The resulting Western blot was probed with mouse anti-p80-coilin. In (**D**), the converse experiment was performed, in which under the same conditions, IP was performed with 5 µg mouse anti-coilin and Western blotting with anti-L4-22K/33K. Note that the images shown of the WCE in panels C and D resulted from shorter exposures than the remainder of the blot, signified by the vertical separating line. (**E,F**) 293T cells were transfected with plasmids encoding Myc-tagged p80-coilin and either Flag-tagged L4-22K or L4-33K. In (**E**), IP was performed with anti-Flag beads (Chromotek, Germany) that interact with the L4 Flag-tagged protein products followed by Western blotting with anti-Myc antibody (to detect any co-precipitated Myc-tagged p80-coilin). In (**F**), the converse experiment was performed, in which anti-Myc-tagged beads were used in immunoprecipitation, followed by Western blotting with anti-Flag antibody to detect co-precipitation of Flag-tagged L4 proteins with Myc-tagged p80-coilin. The Western blots shown are representative of at least three replicate experiments.

The specificity of the polyclonal anti-L4-33K antibody was determined, since the L4-33K and L4-22K proteins share amino acid sequences at the amino-termini of both proteins but have unique carboxy-terminal domains (45, 46). Western blot analysis of whole-cell extracts (WCE) of Ad5-infected A549 cells showed that this antibody recognized two proteins of approx. 35 and 38 kDa that correspond to L4-22K and L4-33K, respectively (Fig. 6A). Their aberrant migration is probably due to the amino acid composition and/or phosphorylation of both proteins (45, 46). The antibody also recognized a protein in mock-infected cells that did not co-migrate with either L4-22K or 33K. Since the antibody recognized both the 22K and 33K proteins, it was termed anti-L4-22K/33K#1 (Fig. 5B and 6A). An independent rabbit polyclonal antibody raised against recombinant L4-33K produced in bacteria also recognized the 22K and 33K proteins (termed anti-L4-22K/33K#2; Fig. 6B) (47). The latter antibody (#2) was also used in Western blotting of extracts of 293T cells that had been transfected with mammalian expression plasmids encoding either the L4-22K or the L4-33K protein. The anti-L4-22K/33K#2 antibody recognized each L4 protein, which co-migrated with the corresponding protein in Ad5-infected A549 cells (Fig. 6B). These results showed significant serological cross-reactivity between the L4-22K and 33K proteins due to their regions of shared amino acid sequence, implying that either L4-22K or L4-33K (or both) co-localized with p80-coilin in microfoci (Fig. 5B,h).

Immunoprecipitation was therefore employed to test whether the L4-22K or L4-33K (or both) proteins formed a stable complex with p80-coilin in Ad5-infected A549 cells (Fig. 6C and D), thus forming a molecular basis for the observed co-localization with p80-coilin. When the anti-L4-22K/33K#2 antibody was used in immunoprecipitation from extracts of Ad5-infected cells, an approx. 80 kDa protein was identified that reacted with anti-p80-coilin in Western blotting (Fig. 6C). This protein species was neither present when control immunoglobulin was used in immunoprecipitation nor when mock-infected cell extracts were similarly analyzed (Fig. 6C). Conversely, when an anti-p80 coilin antibody was used in immunoprecipitation, a Western blot of the immunoprecipitates revealed approx. 33 kDa and 22 kDa proteins when the blot was probed with the anti-L4-22K/33K#2 antibody. However, low levels of the 33 kDa species were evident when control immunoglobulin or extracts from mock-infected cells were used in immunoprecipitation (Fig. 6D). To clarify if the 22K or 33K protein (or both) interacted with p80-coilin, 293T cells were transfected with mammalian expression plasmids encoding myc-tagged human p80-coilin and either FLAG-tagged 22K or 33K. Immunoprecipitation was performed using beads covalently linked to anti-myc or anti-Flag antibodies, and Western blotting was performed on eluates of the beads (Fig. 6E and F). Anti-Flag beads co-precipitated myc-tagged p80-coilin when cells were transfected with 22K-Flag and myc-p80-coilin plasmids but not when 33K-Flag and myc-p80-coilin plasmids were co-transfected (Fig. 6E). Conversely, anti-myc beads co-precipitated 22K-Flag, but not 33K-Flag when myc-p80-coilin and either the 22K-Flag or 33K-Flag plasmids were co-transfected (Fig. 6D).

Taken together, these results show that the L4-22K protein is able to form a stable complex with p80-coilin in Ad5-infected and co-transfected cells. While some evidence was obtained for a complex between L4-33K and p80-coilin in Ad5-infected cells, this was not supported by the co-transfection data and might indicate that another Ad protein may stabilize a putative 33K:p80-coilin complex in Ad-infected cells.

## DISCUSSION

The CB is a sub-nuclear body known to function in snRNP biogenesis (reviewed in reference 16). During infection with Ad5, CBs are fragmented into numerous microfoci arranged into ring-like structures, previously termed “rosettes” (9, 10, 29). In addition, the major protein of CBs, p80-coilin, has been suggested to play a role in the expression of Ad late proteins (IIIa, hexon, and fiber) but not an early protein (E2A-DBP) in HeLa cells (29). In this study, an expanded panel of antibodies against early, intermediate, and late viral proteins and a lung epithelial cell line (A549 cells), frequently used to study respiratory viruses, was used. Depletion of p80-coilin in A549 cells significantly reduced the levels of all Ad5 proteins studied compared to control-infected cells, although the levels of early proteins (E1A and E2A-DBP) were less extensively reduced compared to intermediate (pIX and IVa2) and late proteins. Analysis of Ad5 mRNAs and pre-mRNAs revealed that, for the majority of Ad5 transcripts, p80-coilin depletion did not alter Ad5 transcription or mRNA splicing. However, the comparison of nuclear and cytoplasmic mRNA levels revealed that p80-coilin depletion reduced the transport of Ad5 mRNAs from the nucleus to the cytoplasm. This points to a role for p80-coilin in the nuclear export of Ad mRNAs.

Early and intermediate Ad5 mRNAs are exported from the nucleus via the CRM1 export complex (48), the cellular export pathway for snRNAs, rRNAs, and short-lived, fast-response transcripts such as cytokine mRNAs (49 – 51). In contrast, late Ad5 mRNAs appear to be exported via the Nxf1/TAP export complex (52), which is responsible for the export of the majority of cellular mRNAs (53, 54). As p80-coilin depletion appeared to reduce the export of early, intermediate, and late Ad mRNAs, this suggests that p80-coilin may have roles in both CRM1-mediated and Nxf1/TAP-mediated export of viral mRNAs. Of note, levels of early Ad5 E1A 13S and 12S transcripts and the intermediate transcript pIX in total cellular RNA were reduced following p80-coilin depletion, in contrast to the other 11 Ad mRNAs analyzed. As might be expected, nuclear export of the 13S, 12S, and pIX transcripts was more greatly reduced than the other 11 viral transcripts, with the exception of the E2A-DBP transcripts. It is possible that p80-coilin may have effects on post-transcriptional processing of the E1A 12S and 13S and pIX transcripts other than, or in addition to the modulation of nuclear export, for example, by affecting viral mRNA stability. p80-coilin is involved in snRNA processing (24), and CBs have been implicated in snRNA export (28). As mRNA processing and transport are closely linked (55), it is possible that p80-coilin may be involved in the editing and/or release of mRNAs for export.

During the late phase of Ad infection, the Ad E1B-55K/E4orf6 ubiquitin ligase complex mediates the selective export of late Ad mRNAs, while cellular mRNA export is reduced (56, 57). It is possible that ubiquitination and degradation of an, as yet, unidentified target protein may be required for the selective export of Ad mRNAs. Alternatively, as ubiquitination can also function as a post-translational modification that modifies protein function as well as targeting proteins for degradation (reviewed in reference 58), it is possible that ubiquitination of a cellular protein results in altered activity that switches the export preference from cellular to late Ad mRNAs. In this respect, it would be interesting to establish whether p80-coilin is a ubiquitination target of the E1B-55K/E4orf6 complex during Ad infection. A recent study has shown that certain RNA-binding proteins such as RALY and hnRNP-C are ubiquitinated without overall reduction in their steady-state level during Ad5 infection and may be required for Ad5 E1B-55K/E4orf6-mediated viral RNA export (59). In this study, it was shown that p80-coilin appears to facilitate Ad5 infection and remains stable throughout most of the Ad lifecycle. Therefore, post-translational modification by ubiquitin might be involved in the regulation of viral gene expression rather than as a means of targeting p80-coilin for degradation. The p80-coilin protein is a phosphoprotein (30), and changes in its level of phosphorylation during Ad5 infection may also affect its biological properties.

The mechanism of redistribution of CBs into microfoci during Ad5 infection and the functions of p80-coilin in this process remain to be established. Microfoci appear to occupy a unique location in the nuclei of Ad-infected cells. They are located close to but do not co-localize with viral DNA replication centers (29). It appears that p80-coilin facilitates early, intermediate, and late phase viral mRNA export, yet there is no disassembly of the CB until the intermediate/late phase of infection (29). It is possible that the nucleoplasmic fraction of p80-coilin (that exists outside of the CB) is responsible for viral mRNA export from the nucleus. The majority of p80-coilin is believed to be located in the nucleoplasm (60). Nucleoplasmic p80-coilin has separate functions from snRNP assembly in the CB, including the involvement in DNA damage responses and snRNA editing (24 – 28). Therefore, this lends further support to the notion that it is the nucleoplasmic fraction of p80-coilin that is required for viral mRNA export. Subsequent disassembly of CBs in the late phase of infection could be a consequence of the high level of synthesis of late Ad transcripts (61). CBs may be disassembled at this stage in order to release more p80-coilin into the nucleoplasm to facilitate the export of high levels of late Ad5 transcripts.

In attempting to reveal a link between Ad proteins and p80-coilin, confocal microscopy was performed using a panel of specific antibodies against individual Ad proteins and p80-coilin. The only antibody that produced any co-localization with p80-coilin was raised against the L4-33K protein, which was detected in a fraction of microfoci in the late phase of infection. This might be suggestive of a dynamic association between p80-coilin in microfoci and L4 proteins. There is a further L4 transcript that encodes a 22K protein, whose amino-terminal sequence is identical to that of L4-33K, but differs in its carboxy-terminal domain (7, 45, 46). The anti-L4-33K antibody used in immunofluorescence in this study was generated against the 33K protein derived from Ad-infected HeLa cells. However, Western blot analysis of Ad5-infected A549 cell extracts using this antibody, and an independently produced antibody raised against bacterial recombinant L-33K, showed that both antibodies also recognized L4-22K, presumably due to the recognition of shared amino-terminal domains of both L4 proteins (45, 46). These antibodies were therefore termed anti-L4-22/33K in this study. Therefore, it appeared that either L4-33K, L4-22K, or both proteins co-localized with p80-coilin in microfoci. This prompted a detailed analysis of the interaction between p80-coilin and the L4-22K and L4-33K proteins. Immunoprecipitation of Ad5-infected A549 cell extracts revealed specific complexes between p80-coilin and L4-22K and, to a much lesser extent, L4-33K. Co-transfection of plasmids designed to express epitope-tagged p80-coilin and L4-22K or L4-33K in 293T cells showed clear evidence of a p80-coilin:L4-22K complex, whereas no stable complex could be detected containing L4-33K. These results imply that the interaction site on L4-22K for p80-coilin may lie at the carboxy-terminal domain that is unique to L4-22K. It can be proposed that complex formation between L4-22K and p80-coilin generates the proximity between the two proteins that is the basis for their co-localization in microfoci in Ad5-infected cells. The L4-22K protein has multiple functions in Ad infection, including post-transcriptional regulation of viral gene expression (45, 46). It might be speculated that p80-coilin and L4-22K form a complex involved in the regulation of viral mRNA transport from nuclei to the cytoplasm of Ad-infected cells.

CB redistribution occurs during infection with other viruses. Microfoci-like structures have been observed during infection with the plant RNA virus, groundnut rosette virus (GRV) (61). The GRV movement protein ORF3 appears to utilize CBs to traffic to the nucleolus. Once at the nucleolus, ORF3 associates with fibrillarin, facilitating the incorporation of fibrillarin into viral RNP particles necessary for virus spread (62). A cysteine-rich 16 kDa protein (16K) encoded by the RNA 1 component of tobacco rattle virus (TRV) interacts with tobacco p80-coilin, forming a stable complex demonstrated by co-immunoprecipitation (63). The 16K:p80-coilin complex appears necessary for the recovery of the tobacco plant from TRV infection. While this complex bears some resemblance to the Ad L4-22K:p80-coilin complex, there is no obvious similarity at the amino acid sequence level between 16K and L4-22K or L4-33K (data not shown). Certain human RNA viruses such as Zika and Influenza A virus encode proteins (NS5 and NP, respectively) that target CBs and induce the production of smaller, more numerous CBs in infected cells (34, 64). Among DNA viruses, the NS1 protein of the autonomous mouse parvovirus, minute virus of mice, associates with CBs (65), while the UL3 and UL30 gene products of human cytomegalovirus reduce the number of CBs in infected cells (66). However, the impact of CB targeting on virus replication by many of these mammalian viruses remains to be established.

In conclusion, while a number of studies on several animal and plant viruses have identified CBs as targets in viral infection, there have been few investigations of the functional significance of such interactions between virus proteins and this nuclear substructure of the host cell. This study has revealed a novel function for the major CB protein, p80-coilin, in Ad infection by facilitating the export of viral mRNA transcripts from the nucleus for translation in the cytoplasm. Co-localization of L4-derived viral splicing and transcription factors with a fraction of p80-coilin in microfoci and the discovery of a stable complex between p80-coilin and the L4-22K protein points to a possible role for p80-coilin in coupling the processes of viral mRNA transcription and export.

## MATERIALS AND METHODS

### Cell lines and antibodies

A549 and HeLa cells were obtained from ECACC (Salisbury, UK) and grown in Dulbecco’s minimal essential medium (DMEM) supplemented with 10% (vol/vol) heat-inactivated fetal calf serum (FCS) and 20 mM L-glutamine (termed Complete DMEM) at 37°C in a humidified atmosphere containing 5% CO_2_. A list of all primary and secondary antibodies used is available on request from the corresponding author.

### Virus propagation and infection

Ad5 was propagated in HeLa cells and purified by CsCl gradient purification as previously described (29, 67). Virus titers were determined by the fluorescent focus-forming assay and expressed as focus-forming units (FFU) as previously described (29, 68). A549 cells were seeded in six-well plates and grown to approx. 80% confluence. Ad5 was adsorbed to cells for 1 hour at 37°C at a multiplicity of infection (MOI) of 5 FFU/cell. Complete DMEM was added, and infected cells were incubated for the times indicated in the Figures.

### RNA interference

p80-coilin siRNAs were synthesized by Eurogentec Ltd (Southampton, UK), as previously described (29). AllStars Negative Control siRNAs were purchased from Qiagen UK. A549 cells were grown in six-well plates to approx. 60% confluence in antibiotic-free Complete DMEM, treated with siRNAs in Opti-MEM-1 and Lipofectamine 2000 (Invitrogen, UK) for 4 hours at 37°C according to the manufacturer’s instructions. Transfection medium was replaced with antibiotic-free Complete DMEM, and cells were incubated at 37°C for 24 hours.

### Western blotting

Whole-cell extracts were prepared in RIPA buffer [150 mM NaCl, 1% NP40 substitute, 0.5% DOC, 0.1% SDS, 50 mM Tris-HCl (pH 8.0)] supplemented with 1% protease inhibitor cocktail (Thermo Fisher, UK) for 15 minutes on ice. Protein concentration was determined using the BioRad DC assay. SDS-PAGE and Western blotting were performed as previously described (29). The enhanced chemiluminescence (ECL) detection system was used following the manufacturer’s instructions (GE Healthcare, UK) using a Fuji LAS 3000 Imager, and images analyzed with AIDA software (Raytek, UK).

### Indirect immunofluorescence

A549 cells were grown on glass coverslips and infected as described above. Cells were fixed in 4% paraformaldehyde (Sigma, UK) in PBS for 10 minutes at room temperature, washed in PBS, and permeabilized in PBS containing 1% Triton X-100 for 10 minutes at room temperature. Cells were blocked in PBS containing 10% normal goat serum (NGS; Vector Laboratories, UK) for 10 minutes and incubated with the appropriate primary antibody in PBS plus 1% NGS and 0.1% Triton X-100 for 1 hour at room temperature. Cells were incubated with the appropriate secondary fluorescently conjugated antibody in PBS plus 1% NGS and 0.1% Triton X-100 for 30 minutes in the dark and incubated with 4′−6′-diamidino-2-phenylindole (DAPI) (Sigma, UK) in PBS for 10 minutes in the dark. Coverslips were mounted on slides using Vectashield (Vector Laboratories, UK). Confocal microscopy was performed on an Axioplan inverted confocal microscope (Carl Zeiss, Germany) using LSM Imaging software (Carl Zeiss, Germany).

### Immunoprecipitation

A549 cells were grown in six-well plates and infected by Ad5 viruses as described above; 293T cells were co-transfected with 4 µg of Myc-tagged p80-coilin (a kind gift of Michael Hebert and Madelyn Davis, University of Mississippi Medical Center, USA) and Flag-tagged 33K or 22K expression plasmids (a kind gift of Keith Leppard, University of Warwick, UK). After incubation, cells were washed with ice-cold PBS, and whole-cell lysates were prepared by scraping the cells into 100 µL of ice-cold non-denaturing lysis buffer [20 mM Tris HCl (pH 8), 137 mM NaCl, 1% Triton-X100, 2 mM EDTA], supplemented with protease and phosphatase inhibitor cocktails (Roche, UK). Lysis was performed at 4°C for 30 minutes. Samples were centrifuged at 15,000 × *g* for 10 minutes at 4°C. The protein concentration of the cell lysates was determined using the BioRad DC assay. Aliquots (100 µg) of cell lysates plus 2 µg of antibody (rabbit anti-22/33K or mouse anti-p80 coilin; Santa Cruz, USA) were incubated with gentle rotation at 4°C for 18 hours. The samples were mixed with 20 µL of Sepharose Protein A/G beads (Rockland, USA) and incubated with gentle rotation at 4°C for 4 hours. After incubation, tubes were centrifuged at 3,000 × *g* for 2 minutes at 4°C, the supernatant was removed, and beads were washed with 500 µL of lysis buffer (without protease/phosphatase inhibitor cocktail). The samples were centrifuged, and the washing step was repeated three times. Beads were eluted in 15 µL of 5× SDS loading buffer (250 mM Tris-HCl, pH 6.8, 10% SDS, 5% β-mercaptoethanol, 50% glycerol, and 0.05% bromophenol blue) by heating for 10 minutes at 95°C. Beads were pelleted at 3,000 × *g* for 2 minutes at 4°C, and the supernatants were analyzed by Western blotting as previously described (29). Images from blots were obtained using an ECL advanced chemiluminescence detection kit (Amersham, UK) and a Fuji LAS 3000 luminescent image analyzer (Fuji Life Science, Japan). Images were analyzed using AIDA image analysis software (Raytest, Germany). For cell lysates derived from co-transfected 293T cells, DYKDDDDK Fab-Trap Agarose (Chromotek) and Myc-Trap Agarose (Chromotek) beads were used to capture co-precipitated proteins, according to the manufacturer’s protocol. Anti-FLAG (Cell Signaling, UK) and anti-myc (antibodies.com) antibodies were used in Western blotting.

### RNA isolation, reverse transcription, and qPCR

Whole-cell RNA was prepared using TRIzol reagent, following the manufacturer’s instructions (Invitrogen, UK). For the extraction of nuclear and cytoplasmic RNA, cells were resuspended in ice-cold membrane lysis buffer [50 mM HEPES-KOH (pH 7.5), 150 mM NaCl, 2 mM MgCl_2_, 2 mM CaCl_2_, 1% Triton X-100, 10% glycerol] supplemented with 1% protease inhibitor cocktail (Thermo Fisher, UK) and incubated on ice for 30 minutes. Nuclei were pelleted at 10,000 × *g* for 20 minutes at 4°C. RNA was extracted from the supernatant (cytoplasmic fraction) and nuclear pellet using TRIzol reagent. Reverse transcription of DNAse-treated RNA was performed using random hexamer primers and the Roche Reverse Transcriptor cDNA Kit, according to the manufacturer’s instructions. Quantitative PCR was performed using the Qiagen SYBR Green Kit and the Rotorgene 6000 cycler (Qiagen, UK). Delta-delta Ct values were calculated using Rotorgene 6000 software. A list of all PCR primers used is available on request from the corresponding author.

### Flow cytometry

Cells were detached from wells using trypsin, neutralized in Complete DMEM, pelleted at 350 g for 5 minutes at 4°C prior, and fixed in 10% formalin (Sigma, UK) for 10 minutes. Fixed cells were washed in PBS, permeabilized in PBS containing 1% (vol/vol) Triton-X 100 for 10 minutes, washed in PBS, and blocked in 10% NGS in PBS for 10 minutes. Cells were incubated in primary antibody for 1 hour on ice, washed in PBS, and incubated with the appropriate secondary fluorescent-labeled antibody for 30 minutes at 4°C in the dark. Samples were analyzed on a BD Fortessa flow cytometer (BD Biosciences, New Jersey, USA), and the levels of viral proteins (expressed in geometric mean fluorescence units) were analyzed using FlowJo software (Tree Star Inc, Oregon, USA), as previously described (29).

### Quantitation of infectious Ad5 in siRNA-treated A549 cells

Quantitation of Ad5 produced following infection of control or p80-coilin-depleted A549 cells was performed using serial dilutions of cell lysates either by conventional plaque assay (67) or by quantitation of the major capsid protein, the hexon, by flow cytometry, as described above (29).

### Cell viability (MTT) assay

Stock 3-(4, 5-Dimethylthiazol-2-yl)-2, 5-diphenyltetrazolium bromide (5 mg/mL in PBS; Sigma Aldrich) was diluted to 2.5 mg/mL in Complete DMEM, and 40 µL was added to cells in each well of a 96-well plate. Cells were incubated for 4 hours at 37°C. The medium was removed and cells were washed in PBS. In living cells, the yellow tetrazole MTT was reduced to insoluble purple formazan dye, which was solubilized using 200 µL stock DMSO (Sigma Aldrich) (69). Cells were agitated at room temperature in the dark for 10 minutes. The colored solution was quantified using a spectrophotometer reading at 570 nm. A reading was also taken at 620 nm to account for background.

### Statistical analysis

All data were analyzed using the paired one-sample *t*-test. All experiments were performed at least three times in duplicate.
